# Evaluation of standardized ileal digestibility of amino acids in fermented soybean meal for nursery pigs using direct and difference procedures

**DOI:** 10.5713/ab.22.0269

**Published:** 2022-09-02

**Authors:** Ki Beom Jang, Sung Woo Kim

**Affiliations:** 1 Department of Animal Science, North Carolina State University, Raleigh, NC, 27695, USA

**Keywords:** Amino Acids, Fermented Soybean Meal, Nursery Pigs, Standardized Ileal Digestibility

## Abstract

**Objective:**

This study was to evaluate standardized ileal digestibility (SID) of amino acids (AA) in fermented soybean meal (FSBM) for nursery pigs using both direct procedure and difference procedure when FSBM was added at 20% in diets.

**Methods:**

Forty-eight pigs at 9.2±0.9 kg body weight (BW) were individually housed and allotted to 4 treatments. Treatments included NFD (a semi-purified N free diet), FSD (a diet with 20% FSBM), CBD (corn basal diet), and CFD (corn basal diet:FSBM at 80:20). The FSD was used to measure AA digestibility in FSBM using the direct procedure, whereas CBD and CFD were used in the difference procedure. Pigs were fed for 10 days (0.09×BW^0.75^ kg per day) and euthanized to collect ileal digesta for TiO_2_ and AA.

**Results:**

Total endogenous AA loss was 12.1 g/kg of dry matter intake. The apparent ileal digestibility (AID) Thr was greater (p<0.05) and AID His (p = 0.073) and Leu (p = 0.052) tended to be greater using the direct procedure compared with the difference procedure. The SID Thr were greater (p<0.05) in FSBM for nursery pigs calculated using a direct procedure compared with a difference procedure. In addition, SID Lys in FSBM was about 83% to 88% for nursery pigs higher than SID Lys described in National Research Council (2012).

**Conclusion:**

The SID of AA in FSBM when included at practical levels using the direct procedure were similar to those from the difference procedure. Considering the SID of AA obtained using both direct and difference procedures, FSBM is an effective protein supplement providing highly digestible AA to nursery pigs. The SID of AA from this study was considerably higher than those previous reported. This study also indicates the importance of including the test feedstuffs at practical levels when evaluating digestibility.

## INTRODUCTION

Soybean meal has various anti-nutritional factors including trypsin inhibitors, lectins, glycinin, and β-conglycinin possibly causing negative impacts on growth and nutrient digestion of pigs [1–3]. Various processing for soybean meal has been developed to eliminate or reduce the anti-nutritional factors in soybean meal. Fermented soybean meal (FSBM) provides plant-based protein after microbial fermentation to reduce the anti-nutritional factors. Fermented soybean meal contained the lower concentrations of glycinin, β-glycinin, and trypsin inhibitors compared conventional soybean meal [2,3]. Previous studies has also been shown that FSBM positively affects not only growth performance and protein digestibility [3–5], but also inflammatory response and antioxidant activity of nursery pigs compared with conventional soybean meal [6].

Availability of amino acids (AA) in FSBM has been re ported in previous literature. Interestingly, there is substantial difference on the standardized ileal digestibility (SID) AA (SID Lys; 75% vs 86%) in FSBM for growing pigs between NRC [7] and the latest report [8]. Standardized ileal digestibility of AA in FSBM reported in NRC [7] is based on two observations and the digestibility did not consider the growth stages of pigs. Other published studies reporting SID AA in FSBM for nursery pigs used a direct procedure with at least 30% FSBM [4,5,9–12]. The direct procedure uses a diet mainly with a test feedstuff and other minor feedstuffs that are fully digestible nutrients so that the digestibility of interested nutrients are mainly contributed from the test feedstuff. In direct procedure, the use of a test feedstuff is often greater than practical inclusion levels included in the diet [4,5,10]. Considering that practical inclusion levels of FSBM in nursery feeds are between 3% to 20% [3,9,12], SID AA measured in a diet containing 30% FSBM could underestimate its digestibility due to palatability or antinutritional factors at increased levels [13,14]. The difference procedure can be applied to estimate digestibility of a test feedstuff at practical inclusion levels as other typical feedstuffs can be used in a test diet [14]. In the difference procedure, a test feedstuff replaces a basal diet at one or several designated proportions and then the digestibility of nutrients in the test feedstuff is calculated considering the contribution of digestibility from a basal diet [13–15]. A basal diet is composed of only a few feedstuffs that are well characterized for their digestibility. According to Adeola [16], the procedure error would be related to the replacement levels of a test feedstuff in test diets because the nutrient digestibility in a feedstuff is estimated based on extrapolation to 100% replacement of interested nutrient against the replacement levels. Thus, the AA digestibility in FSBM for nursery pigs should be estimated with consideration the practical inclusion levels in test diets for both the direct procedure and the difference procedure. It is, therefore, hypothesized that SID AA in FSBM for nursery pigs should be evaluated through both direct procedure and difference procedure with consideration of a practical inclusion level of FSBM in swine diets. Therefore, the objective of this study was to evaluate SID of AA in FSBM for nursery pigs by using both direct procedure and difference procedure when FSBM is added at 20% in testing diets fed to nursery pigs.

## MATERIALS AND METHODS

### Animal care

The experimental protocol was approved by the Institutional Animal Care and Use Committee of North Carolina State University.

### Animals, experimental design, and diets

A total of 48 newly weaned pigs (24 barrows and 24 gilts) at 21 d of age fed a common diet (26% crude protein [CP] and 15.2% lactose) for 7 days. Nursery pigs at 9.24±0.90 kg body weight (BW) were allotted to 4 treatments with 12 replications per diet in a randomized complete block design with sex and initial BW as blocks. Treatments were NFD (a semi-purified N free diet to measure basal endogenous AA losses), FSD (a diet with 20% FSBM [Pepsoygen; Nutraferma Inc., North Sioux City, SD, USA]), CBD (a corn basal diet), and CFD (a corn basal diet:FSBM = 80:20). Four experimental diets were formulated (Tables 1, 2). Two diets contained corn or FSBM (Pepsoygen; Nutraferma Inc., USA), respectively, as the source of AA and another diet was formulated by adding a certain amount of FSBM into the CBD diet for a mixture of corn and FSBM diet that containing 80% of CBD and 20% FSBM as described by Adeola [16]. A NFD based on corn starch and sucrose was also prepared to measure the endogenous losses of AA. Experimental diets were supplemented with 0.4% titanium dioxide (TiO_2_) as an indigestible marker. Vitamins and minerals were included in the diets to meet or exceed requirement estimates [7]. Pigs were individually housed in each pen that were equipped with a feeder and a nipple drinker.

### Feeding and sample collection

Pigs were fed the experimental diets for 10 d. During the following period, the daily feed allowance was approximately 0.09×BW^0.75^ kg was provided to the animals as 2 equal meals at 08:00 and 17:00. Pigs had free access to water. After 10 d feeding, all pigs were euthanized at the end of the experiments to collect at least 30 mL ileal digesta samples. Ileal digesta were stored at −20°C immediately after ileal digesta collection.

### Chemical analysis

The frozen ileal digesta samples were dried in a freeze dryer. Ingredient and diet samples were analyzed for dry matter (DM; method 930.15) and CP (method 990.03). Diet samples were also analyzed for calcium (method 978.02), phosphorus (method 946.06), neutral detergent fiber (method 2002.04), and acid detergent fiber (method 973.18) as described in AOAC [17]. Amino acid concentrations of diets and ileal digesta samples were determined by ion-exchange chromatography with post-column derivatization with ninhydrin. Before analysis, samples were liberated from the proteiADFn by hydrolysis with 6 *N* HCl for 24 h at 110°C (method 982.30). Methionine and cystine were analyzed as methionine sulfone and cysteic acid after cold performic acid oxidation overnight before hydrolysis. Tryptophan was determined after NaOH hydrolysis for 22 h at 110°C. The concentrations of titanium dioxide in diets and ileal digesta samples were determined by the procedures as previously described by Myers et al [18].

### Calculation and statistical analysis

The apparent ileal digestibility and the true ileal digestibility of AA were calculated for all diets except the NFD, and endogenous losses of AA were calculated from pigs fed NFD as follows Kong and Adeola [13].


$$\begin{array}{l} \text{Apparent ileal digestibility }(\text{AID})=1-({\text{AA}}_{\text{i}}/{\text{AA}}_{\text{d}})\times ({\text{Ti}}_{\text{d}}/{\text{Ti}}_{\text{i}})\\ \text{Basal endogenous losses }(\text{BEL})\;(\text{g}/\text{kg DMI})={\text{AA}}_{\text{i}}\times ({\text{Ti}}_{\text{d}}/{\text{Ti}}_{\text{i}})\\ \text{SID}=[\text{AID}+(\text{BEL}/{\text{AA}}_{\text{d}})] \end{array}$$

where Ti_d_ and Ti_i_ represent the concentration of Ti (g/kg DM) in diets and ileal digesta, respectively. The concentrations of AA (g/kg DM) in diets and ileal digesta represent AA_d_ and AA_i_, respectively. The ileal digestibility of AA in FSBM from the diets that contained both corn and FSBM were also calculated by difference procedure [13].


$$\begin{array}{l} {\text{AID}}_{\text{FSBM}}\;(\%)=[({\text{AID}}_{\text{Mixed}}\times {\text{AA}}_{\text{Mixed}})-({\text{AID}}_{\text{Corn}}\times {\text{AA}}_{\text{Corn}})]/{\text{AA}}_{\text{FSBM}}\\ {\text{SID}}_{\text{FSBM}}\;(\%)=[({\text{SID}}_{\text{Mixed}}\times {\text{AA}}_{\text{Mixed}})-({\text{SID}}_{\text{Corn}}\times {\text{AA}}_{\text{Corn}})]/{\text{AA}}_{\text{FSBM}} \end{array}$$

where AA_corn_ and AA_Mixed_ represent the concentration of AA (g/kg DM) in corn and corn and FSBM mixed diets, respectively. AID_corn_ or SID_corn_ and AID_Mixed_ or SID_Mixed_ are measured AID or SID of AA in corn and corn and FSBM mixed diets, respectively.

Data were analyzed using the Proc Mixed procedure of SAS (SAS Inst. Inc., Cary, NC, USA). In the statistical model, diet included as a fixed variable and a block and sex were the random variables for ileal digestibility in respective diets. Least squares of means for each treatment were calculated. For the comparison between measured and predicted AID and SID of AA in FSBM, the predicted digestibility was subtracted from the measured digestibility and the difference between measured and predicted values in FSBM were tested using a t-test. The statistical difference was considered significant with p<0.05, whereas 0.05≤p<0.10 was considered as tendency.

## RESULTS

Amino acid composition in corn and FSBM used in this study is described in Table 3. The Lys, Thr, Trp, and Met were 0.26%, 0.27%, 0.06%, and 0.17% in corn and 3.16%, 2.11%, 0.61%, and 0.75% in FSBM, respectively. During the experiment, one pig fed diet based on NFD and two pigs fed diet based on FSBM were removed from the 9 experiment due to unnormal healthy conditions. The AID of AA in pigs fed FSD were higher (p<0.05) compared with the pigs fed CBD (Table 4). Endogenous AA losses were determine using pigs fed the NFD and average total 12.1 g/kg of dry matter intake (DMI) (Table 5). The SID of most AA in pigs fed FSD were higher (p<0.05) in the pigs compared with pigs fed CBD and CFD (Table 6).

There was no difference on the AID of Arg, Ile, Lys, Met, Phe, Trp, and Val in indispensable AA and Asp, Gly, Pro, Ser, and Tyr in dispensable AA of FSBM between direct and difference procedures (Table 7). The AID of Thr was greater (p<0.05) and AID of His (p = 0.073) and Leu (p = 0.052) tended to be greater if calculated using the direct procedure rather than difference procedure. The AID of Ala, Cys, and Ser were greater and the AID of Glu tended to be greater (p = 0.061) if calculated using the direct procedure rather than difference procedure.

There was no difference on the SID of most indispensable AA except for Thr (p<0.05) of FSBM between direct and difference procedures (Table 8). The SID of dispensable AA including Ala and Ser in FSBM were greater (p<0.05) and SID of dispensable AA including Cys (p = 0.054) and Gly (p = 0.079) in FSBM tended to be greater if calculated using the direct procedure rather than difference procedure.

## DISCUSSION

Amino acids are responsible for the most parts of physiological responses for growth and maintenance in the body such as nutrient metabolism, immunity, and protein deposition [19,20]. In particular, nursery pigs would be susceptible from multiple stressors during postweaning periods, leading to reduced feed intake and growth retardation with impaired intestinal functions, especially barrier function and nutrient utilization [21,22]. According to previous studies, AA balance and some specific AA could be significantly engaged in the modulation of immunity and recovery the intestinal damage induced from weaning in nursery pigs [23–25]. Thus, estimation of specific AA availability in feedstuff would be important to efficiently supply and utilize the AA for growth and health of nursery pigs.

Soybean meal is the main protein supplement in swine feeds, but it has been used at less than 20% in nursery feeds because the immature intestine of nursery pigs do not tolerate to digest the high levels of soybean meal [2,26,27]. Fermented soybean meal could be an effective source of soy protein and it is produced by mixing conventional soybean meal with water and a bacterial culture achieved from human food production, microbial fermentation, and then drying it on a plate or drum drier at a specific temperature to avoid heat damage to the protein [28]. Microbial fermentation by beneficial microbes such as *Apergillus oryzae* and *Bacillus subtillits* could effectively reduce anti-nutritional factors such as the trypsin inhibitor by 84%, glycinin by 40%, beta-conglycinin by 40%, and phytic acid by 35% with increasing protein content and producing smaller peptide size in conventional soybean meal [2,3,28]. Previous studies have shown that supplementation of FSBM positively affects growth performance and protein digestibility of nursery pigs compared with conventional soybean meal [3–5].

In this study, the analyzed compositions of indispensable and dispensable AA in FSBM were similar or slightly higher than those in conventional soybean meal described in NRC [7]. These difference would be possibly due to the inclusion rate of soybean hulls during the fermentation process, which may affect the AA concentrations in FSBM [29]. Microbial fermentation in soybean meal could decrease the composition of DM contents and trypsin inhibitors with increase in smaller peptide and fat contents and it would be accounted for by carbohydrate fermentation by *Apergillus oryzae* [2]. Thus, the use of fermented soybean would be beneficial to efficiently supply high AA through changing the AA profiles and reducing the antinutritional factors by microbial fermentation compared with conventional soybean meal.

In this study, the SID of AA in CBD and CFD diets for nursery pigs were relatively low compared with the SID of AA reported in previous studies. The CBD and CFD diets in this study were used to estimate the SID of AA in FSBM using a difference procedure. In contrast to the SID of AA in FSD, which was relatively similar to the SID of AA from previous literature [8,9,30], the possible reason for this observation would be related to the inclusion of corn as a major feedstuff in CBD and CFD diets. According to Oliveira et al [15], the SID of AA in the corn-basal diet and corn-SBM diet was over 85% for growing pigs. However, considering the growth phase of pigs, Trindade Neto et al [31] showed that the SID of AA in corn for nursery pigs was relatively low with the SID of most AA at below 30%. Sauer et al [32] also showed that the SID of AA in corn for nursery pigs was also relatively similar to the SID of AA in this study. It can be explained by the high variable digestibility of AA in corn [33–35]. However, the possible reason for this observation could also be related to the capacity of nursery pigs to digest the plant feedstuff during the post-weaning period, although corn is one of the most used cereals as an energy source containing less antinutritional factors. According to Mahan [36], starch from cereal grains is low palatable and less digestible for newly weaned pigs rather than lactose, because their digestive tract would be adapted to lactose digestion by milk consumption during lactation. Moeser et al [37] showed that intestinal damage by weaning stress would be sustained for about 14 d after weaning with impaired intestinal functions, resulting in a reduction in the digestive and absorptive capacity in the small intestine of nursery pigs. Lindemann et al [38] also showed the activities of digestive enzymes in nursery pigs could be reduced during the first 2 wks after weaning. In this study, although the pigs had an adaptation period for 10 d after weaning, their intestine would be limited to digesting the corn as a plant feedstuff in CBD and CFD diets. Therefore, the results in this study indicate that the inclusion level of a test feedstuff that affects the inclusion of other typical feedstuffs such as corn in test diets for a difference procedure could be important to determine the SID of AA in feedstuff for nursery pigs.

Amino acid digestibility in feed ingredients have been de termined either by direct procedure or difference procedure [13,14]. The direct procedure can be used for determining digestibility of nutrients in the feed ingredients with high feeding value such like palatable feedstuff and low content of antinutrients [13,14]. In difference procedure, when the test feedstuff would not be solely used as the major ingredient due to poor palatability, high content of protein, or anti-nutritional factors. For a difference procedure, testing diets are required to be formulated with both the test feedstuff and cereal feedstuff. A direct procedure has been widely used to evaluate the SID of protein feedstuff rather than a difference procedure [4,5,8,9,15], because having poor palatability or high content of proteins and antinutritional factors can interrupt the evaluation of AA digestibility in a feedstuff using a difference procedure [13]. Based on the findings in previous studies [2,3,28], it could suggest that FSBM at a practical inclusion level can have additivity of SID AA because microbial fermentation leads to the change in protein profile and the reduction of the concentration of antinutritional compounds in conventional soybean meal. The inclusion level of soy protein source below 20% in early weaner diets also has been considered the palatability and digestive capacity of nursery pigs [2,26,27].

In this study, the AID of dispensable AA including His, Leu, and Thr in FSBM from direct procedure were less than from difference procedure, but the SID of AA in FSBM were not different except for Thr. The possible reason for the non-additivity on AID and SID of these AA would be related to the contribution of digestible AA in corn for a difference procedure. This study considered the practical inclusion level of FSBM in nursery feeds and thus the experiment diets contained the relatively lower inclusion levels of FSBM at 20% in the testing diets compared with previous studies using about 30% [4,5,8–10,12]. It could also bring an increase in the portion of the use of corn in the test diets for a difference procedure. This may indicate that digestibility of AA in corn could result in relatively high variation in AID and SID of AA in corn and thus it may affect the estimation for a difference procedure to measure AID and SID of several AA in FSBM for nursery pigs. Previous studies have shown the cereal grains including corn could have high variable digestibility of AA [33–35] that were not also in accordance with NRC [7]. According to Stein et al [33], the AID of AA in feedstuff could not be additive depending on the AA composition in the testing diets. Stein et al [39] also showed that the low concentration of AA in feed ingredients may be potential to cause variation on the AID of AA due to the relatively greater contribution of AA of endogenous origin to the ileal output of AA in feed ingredients. Xue et al [40] also showed that the predicted AID of AA based on the values from mixed diets containing a high proportion of corn as low CP feedstuff be likely to be lower than the determined values by a direct method. Therefore, an increase in the portion of corn in the testing diets for a difference procedure may influence to be underestimated values for the AID and SID of several AA in FSBM using a difference procedure and thus resulted in the lack of additivity.

Most of the previous literature reported the SID of AA in FSBM for pigs by a direct procedure [4,5,8,9], but there is limited information about those from a difference procedure. Although this study shows the SID of AA in FSBM using both a direct procedure and a difference procedure, comparing the results with literature within the same method would be necessary. Comparing the SID of AA in previous reports with consideration of the type of FSBM, the BW of pigs, and the measuring procedure, interestingly, the SID of Lys in FSBM for pigs was lower than the values in conventional soybean meal. Cervantes-Pahm and Stein [4] also showed 9% lower SID Lys in FSBM with *Aspergillus oryzae* (77% vs 85%) than in conventional soybean meal for nursery barrows during 10.9 to 22.2 kg BW. The SID Lys in FSBM was 16% lower (75% vs 89%) than in conventional soybean meal in NRC [7]. The SID Lys in FSBM with *Aspergillus oryzae* and *Bacillus subtilis* was 2% lower (82% vs 84%) for nursery pigs at 10.4 kg initial BW in Rojas and Stein [5]. The SID Lys in FSBM with *Streptococcus thermophiles* and *Saccharomyces cerevisiae* was 5% (83% vs 88%) lower for growing barrows at 26.8 kg initial BW in Wang et al [9]. The SID Lys in FSBM with *Aspergillus oryzae* and *Bacillus subtilis* was 4% lower (86% vs 90%) for grower-finisher barrows at 30.4 kg initial BW in Yáñez et al [8]. Cervantes-Pahm and Stein [4] demonstrated that the reduced the SID of lysine would be results from the heating processing in FSBM production which is possibly causing the Maillard reaction with decreased the available Lys content. Besides, based on previous findings, the SID of AA in FSAM was not related to the BW of pigs or the procedure, because SID of AA in various types of FSBM would be influenced by the bacterial species as previously reported by Kim et al [11]. Indeed, the SID Lys in FSBMs seems to be getting increased as times go on. Based on the published years of studies, the initial studies showed underrated SID Lys for pigs compared with the latest studies. This study also shows that the SID Lys in FSBM was about 83 (difference procedure) to 88% (direct procedure) for nursery pigs. These values are relatively accordance in the values from the latest reports [8,9,30] rather than the initial reports [4,7,10]. It may be possibly due to the advances in the processing technology using microbial fermentation by specific bacteria to consider the impacts on available AA content in soybean meal for growing pigs [8,11,41]. Unfortunately, conventional soybean meal was not tested in this study indicating that it would not be available to directly comparison in the AA digestibility between FSBM and conventional soybean meal. Another possible reason could be suggested by the inclusion levels of FSBM in testing diets. Previous studies using a direct procedure with around 30% FSBM showed relatively low SID Lys (average 77%) in FSBM for nursery pigs [4,5,10]. In contrast, previous studies using a direct procedure with around 25% FSBM showed SID Lys at an average of 84% in FSBM for nursery pigs [9,12]. Due to possibly causing the low palatability or the content of antinutritional factors when a test feedstuff is added at high levels in test diets [13,14], the SID AA in FSBM would be variable by the inclusion level of FSBM in experimental diets.

## CONCLUSION

In conclusion, the SID of AA in FSBM when included at practical levels using the direct procedure were relatively similar to those from the difference procedure. Considering SID of AA obtained using both direct procedure and difference procedure, FSBM is an effective protein supplement providing highly digestible AA to nursery pigs. The SID of AA from this study was considerably higher than those previous reported. This study also indicates the importance of including the test feedstuffs at practical levels when evaluating digestibility.

## Figures and Tables

**Table 1:** Composition of experimental diets^1)^ (as-fed basis)

Item	FSD	CBD	NFD
Feedstuff (%)			
Fermented soybean meal	20.00	-	-
Corn, yellow	-	92.62	-
Corn starch	62.72	-	69.37
Sucrose	10.00	-	20.00
Cellulose	3.50	-	5.00
Soy oil	-	3.00	1.00
L-Lys HCl	-	0.38	-
L-Thr	-	0.08	-
L-Trp	-	0.04	-
Vitamin premix^2)^	0.03	0.03	0.03
Mineral premix^3)^	0.15	0.15	0.15
Magnesium oxide	-	-	0.10
Potassium carbonate	-	-	0.40
Salt	0.40	0.30	0.40
Dicalcium phosphate	2.00	2.35	2.60
Limestone	0.70	0.55	0.45
Titanium dioxide	0.50	0.50	0.50
Total	100.00	100.00	100.00

1) FSD, a diet with 20% fermented soybean meal (Pepsoygen, Nutraferma Inc., North Sioux City, SD, USA); CBD, a corn basal diet; NFD, a semi-purified N free diet to measure basal endogenous AA losses; CFD, a mixture of corn and fermented soybean meal diet (80% of CBD and 20% fermented soybean meal).

2) The vitamin premix provided per kilogram of complete diet: 6,614 IU of vitamin A as vitamin A acetate, 992 IU of vitamin D3, 19.8 IU of vitamin E, 2.64 mg of vitamin K as menadione sodium bisulfate, 0.03 mg of vitamin B_12_, 4.63 mg of riboflavin, 18.52 mg of D-pantothenic acid as calcium panthonate, 24.96 mg of niacin, and 0.07 mg of biotin.

3) The trace mineral premix provided per kilogram of complete diet: 33 mg of Mn as manganous oxide, 110 mg of Fe as ferrous sulfate, 110 mg of Zn as zinc sulfate, 16.5 mg of Cu as copper sulfate, 0.30 mg of I as ethylenediamine dihydroiodide, and 0.30 mg of Se as sodium selenite.

**Table 2:** Analyzed composition of experimental diets^1)^ (as-fed basis)

Item	FSD	CBD	CFD	NFD
DM (%)	95.56	92.38	91.41	95.71
CP (%)	10.83	7.26	17.33	0.44
NDF (%)	5.24	8.00	7.46	4.95
ADF (%)	4.30	2.62	3.09	4.53
Ca (%)	0.72	0.75	0.61	0.71
P (%)	0.49	0.73	0.67	0.52
Indispensable AA (%)				
Arg	0.69	0.37	1.06	0.01
His	0.28	0.20	0.45	0.00
Ile	0.55	0.26	0.78	0.01
Leu	0.89	0.79	1.53	0.01
Lys	0.68	0.54	1.14	0.03
Met	0.15	0.16	0.29	0.00
Phe	0.58	0.34	0.87	0.01
Thr	0.43	0.33	0.70	0.01
Trp	0.15	0.10	0.23	0.00
Val	0.59	0.35	0.88	0.01
Dispensable AA (%)				
Ala	0.51	0.50	0.91	0.01
Asp	1.27	0.49	1.70	0.01
Cys	0.16	0.15	0.31	0.00
Glu	2.02	1.23	2.98	0.02
Gly	0.50	0.31	0.76	0.01
Pro	0.55	0.58	1.02	0.03
Ser	0.50	0.29	0.72	0.01
Tyr	0.23	0.22	0.51	0.01
Total AA	11.06	7.52	17.15	0.44

DM, dry matter; CP, crude protein; NDF, neutral detergent fiber; ADF, acid detergent fiber; AA, amino acids.

1) FSD, a diet with 20% fermented soybean meal (Pepsoygen, Nutraferma Inc., North Sioux City, SD, USA); CBD, a corn basal diet; CFD, a mixture of corn and fermented soybean meal diet (80% of CBD and 20% fermented soybean meal); NFD, a semi-purified N free diet to measure basal endogenous AA losses.

**Table 3:** Analyzed composition of test ingredients (as-fed basis)

Item (%)	Corn	Fermented soybean meal
Indispensable AA		
Arg	0.40	3.57
His	0.22	1.29
Ile	0.28	2.30
Leu	0.85	4.09
Lys	0.26	3.16
Met	0.17	0.75
Phe	0.37	2.71
Thr	0.27	2.11
Trp	0.06	0.61
Val	0.38	2.55
Dispensable AA		
Ala	0.54	2.37
Asp	0.53	5.91
Cys	0.16	0.97
Glu	1.33	9.32
Gly	0.33	2.34
Pro	0.63	2.97
Ser	0.31	2.74
Tyr	0.24	1.74

AA, amino acids.

**Table 4:** Apparent ileal digestibility of amino acids in experimental diets

Item (%)	Treatment^1)^			SEM	p-value

	FSD	CBD	CFD		
Indispensable AA					
Arg	84.6^c^	55.5^a^	73.8^b^	2.9	<0.001
His	86.0^c^	44.9^a^	61.9^b^	3.3	<0.001
Ile	85.0^c^	37.7^a^	64.3^b^	3.8	<0.001
Leu	83.8^b^	50.5^a^	58.6^a^	4.5	<0.001
Lys	81.6^b^	65.3^a^	69.3^a^	3.4	<0.001
Met	85.4^b^	60.3^a^	63.9^a^	4.7	<0.001
Phe	85.4^c^	44.0^a^	62.8^b^	3.8	<0.001
Thr	72.9^c^	29.2^a^	45.0^b^	4.9	<0.001
Trp	80.7^c^	51.9^a^	64.6^b^	3.6	<0.001
Val	79.1^c^	30.5^a^	53.0^b^	3.9	<0.001
Dispensable AA					
Ala	77.0^b^	44.1^a^	48.2^a^	4.1	<0.001
Asp	83.4^c^	33.4^a^	63.6^b^	3.1	<0.001
Cys	73.1^b^	36.1^a^	43.8^a^	4.6	<0.001
Glu	85.9^c^	51.6a	65.6^b^	3.3	<0.001
Gly	42.6^c^	−55.7^a^	5.4^b^	15.3	<0.001
Pro	56.7	38.9	48.9	6.5	0.128
Ser	79.8^c^	31.6^a^	53.5^b^	3.9	<0.001
Tyr	74.6^c^	40.7^a^	58.6^b^	4.9	<0.001
Total AA	79.3^c^	40.8^a^	57.0^b^	3.5	<0.001

SEM, standard error of the mean; AA, amino acids.

1) FSD, pigs fed a diet with 20% fermented soybean meal (Pepsoygen, Nutraferma Inc., North Sioux City, SD, USA); CBD, pigs fed a corn basal diet; CFD, pigs fed a mixture of corn and fermented soybean meal diet (80% of CBD and 20% fermented soybean meal).

a–c Means in the same row with different superscripts are different (p<0.05).

**Table 5:** Basal endogenous losses of amino acids of nursery pigs fed an N-free diet

Item (g/kg dry matter intake)	Basal endogenous losses of AA	SEM
Indispensable AA		
Arg	0.56	0.09
His	0.16	0.02
Ile	0.32	0.06
Leu	0.53	0.10
Lys	0.42	0.09
Met	0.09	0.02
Phe	0.32	0.06
Thr	0.52	0.07
Trp	0.13	0.02
Val	0.55	0.07
Dispensable AA		
Ala	0.54	0.08
Asp	0.74	0.12
Cys	0.14	0.02
Glu	0.97	0.18
Gly	1.93	0.21
Pro	3.09	0.83
Ser	0.45	0.05
Tyr	0.23	0.04
Total AA	12.12	1.66

AA, amino acids; SEM, standard error of the mean.

**Table 6:** Standardized ileal digestibility of amino acids in experimental diets

Item (%)	Treatment^1)^			SEM	p-value

	FSD	CBD	CFD		
Indispensable AA					
Arg	91.7^b^	80.7^a^	79.9^a^	3.1	0.005
His	90.3^b^	61.4^a^	65.1^a^	3.4	<0.001
Ile	89.7^b^	62.6^a^	69.0^a^	3.9	<0.001
Leu	88.8^b^	65.9^a^	62.1^a^	4.7	<0.001
Lys	87.1^b^	81.5^ab^	74.0^a^	3.4	0.005
Met	90.0^b^	72.5^a^	66.7^a^	5.0	<0.001
Phe	90.1^b^	64.2^a^	66.9^a^	4.7	<0.001
Thr	83.6^b^	59.1^a^	53.3^a^	4.9	<0.001
Trp	89.0^b^	76.5^a^	71.7^a^	3.6	<0.001
Val	87.3^b^	60.2^a^	60.0^a^	4.2	<0.001
Dispensable AA					
Ala	85.7^b^	64.6^a^	54.1^a^	4.3	<0.001
Asp	88.1^b^	63.0^a^	68.7^a^	3.2	<0.001
Cys	80.2^b^	57.5^a^	48.5^a^	5.0	<0.001
Glu	89.9^b^	68.9^a^	69.2^a^	3.3	<0.001
Gly	81.1^b^	43.5^a^	35.6^a^	15.2	0.013
Pro	104.8^b^	100.2^ab^	78.3^a^	7.6	0.023
Ser	87.1^b^	59.9^a^	60.0^a^	4.1	<0.001
Tyr	84.3^c^	50.5^a^	62.9^b^	4.5	<0.001
Total AA	89.8^b^	55.7^a^	63.6^a^	3.4	<0.001

SEM, standard error of the mean; AA, amino acids.

1) FSD, pigs fed a diet with 20% fermented soybean meal (Pepsoygen, Nutraferma Inc., North Sioux City, SD, USA); CBD, pigs fed a corn basal diet; CFD, pigs fed a mixture of corn and fermented soybean meal diet (80% of CBD and 20% fermented soybean meal).

a–c Means in the same row with different superscripts are different (p<0.05).

**Table 7:** Apparent ileal digestibility of amino acids in fermented soybean meal calculated using direct and difference procedures

Item (%)	Direct procedure	Difference procedure	Pooled SEM	p-value
Indispensable AA				
Arg	84.1	84.9	7.4	0.919
His	86.0	73.3	6.0	0.073
Ile	84.0	87.3	6.8	0.646
Leu	83.0	64.0	8.1	0.052
Lys	81.5	75.1	9.0	0.498
Met	84.0	66.6	10.2	0.131
Phe	84.7	73.9	6.4	0.136
Thr	73.3	44.3	9.4	0.017
Trp	81.0	78.5	6.9	0.732
Val	78.9	67.0	8.7	0.212
Dispensable AA				
Ala	76.7	45.3	7.9	0.005
Asp	83.4	75.3	6.4	0.244
Cys	73.1	34.2	13.5	0.024
Glu	85.9	73.1	5.8	0.061
Gly	45.0	24.8	16.4	0.260
Pro	56.7	43.8	29.4	0.674
Ser	79.9	50.4	6.8	0.003
Tyr	73.6	59.1	12.4	0.282

SEM, standard error of the mean; AA, amino acids.

**Table 8:** Standardized ileal digestibility of amino acids in fermented soybean meal calculated using direct and difference procedures

Item (%)	Direct procedure	Difference procedure	Pooled SEM	p-value
Indispensable AA				
Arg	91.8	90.0	7.4	0.818
His	91.5	83.3	6.0	0.217
Ile	89.7	95.3	6.8	0.437
Leu	88.6	73.7	8.1	0.107
Lys	87.5	83.3	9.0	0.656
Met	89.8	75.1	10.2	0.191
Phe	90.0	81.7	6.4	0.237
Thr	84.9	59.1	9.4	0.028
Trp	89.3	90.4	6.9	0.874
Val	87.8	78.6	8.7	0.323
Dispensable AA				
Ala	86.8	59.4	7.9	0.010
Asp	89.0	83.5	6.4	0.420
Cys	81.7	50.5	13.5	0.054
Glu	90.5	80.7	5.8	0.131
Gly	81.8	48.0	16.4	0.079
Pro	110.4	66.0	29.4	0.175
Ser	88.5	59.9	6.8	0.004
Tyr	83.3	68.1	12.4	0.262

SEM, standard error of the mean; AA, amino acids.
